# “Health divide” between indigenous and non-indigenous populations in Kerala, India: Population based study

**DOI:** 10.1186/1471-2458-12-390

**Published:** 2012-05-29

**Authors:** Slim Haddad, Katia Sarla Mohindra, Kendra Siekmans, Geneviève Màk, Delampady Narayana

**Affiliations:** 1 CRCHUM, Centre de Recherche du Centre Hospitalier de l’Université de Montréal, 3875, Avenue Saint Urbain, Montréal, Québec, H2W 1V1, Canada; 2 Institute of Population Health, University of Ottawa, Ottawa, Ontario, K1N 6N5, Canada; 3 Centre for Development Studies, Prasanth Nagar, Ulloor, Thiruvananthapuram, 695 011, Kerala, India

## Abstract

**Background:**

The objective of this study is to investigate the magnitude and nature of health inequalities between indigenous (Scheduled Tribes) and non-indigenous populations, as well as between different indigenous groups, in a rural district of Kerala State, India.

**Methods:**

A health survey was carried out in a rural community (N = 1660 men and women, 18–96 years). Age- and sex-standardised prevalence of underweight (BMI < 18.5 kg/m^2^), anaemia, goitre, suspected tuberculosis and hypertension was compared across forward castes, other backward classes and tribal populations. Multi-level weighted logistic regression models were used to estimate the predicted prevalence of morbidity for each age and social group. A Blinder-Oaxaca decomposition was used to further explore the health gap between tribes and non-tribes, and between subgroups of tribes.

**Results:**

Social stratification remains a strong determinant of health in the progressive social policy environment of Kerala. The tribal groups are bearing a higher burden of underweight (46.1 vs. 24.3%), anaemia (9.9 vs. 3.5%) and goitre (8.5 vs. 3.6%) compared to non-tribes, but have similar levels of tuberculosis (21.4 vs. 20.4%) and hypertension (23.5 vs. 20.1%). Significant health inequalities also exist within tribal populations; the Paniya have higher levels of underweight (54.8 vs. 40.7%) and anaemia (17.2 vs. 5.7%) than other Scheduled Tribes. The social gradient in health is evident in each age group, with the exception of hypertension. The predicted prevalence of underweight is 31 and 13 percentage points higher for Paniya and other Scheduled Tribe members, respectively, compared to Forward Caste members 18–30 y (27.1%). Higher hypertension is only evident among Paniya adults 18–30 y (10 percentage points higher than Forward Caste adults of the same age group (5.4%)). The decomposition analysis shows that poverty and other determinants of health only explain 51% and 42% of the health gap between tribes and non-tribes for underweight and goitre, respectively.

**Conclusions:**

Policies and programmes designed to benefit the Scheduled Tribes need to promote their well-being in general but also target the specific needs of the most vulnerable indigenous groups. There is a need to enhance the capacity of the disadvantaged to equally take advantage of health opportunities.

## Background

Large inequalities in health exist between indigenous and non-indigenous populations worldwide [1]. This “health divide” has also been demonstrated in India [2], where indigenous groups are officially classified as Scheduled Tribes. Compared to national averages, Scheduled Tribes have higher mortality rates [3] and experience a greater prevalence of tuberculosis [4] and undernutrition [5], including high anaemia levels [6,7]. These groups are also exposed to higher risks of inadequate food intake [8], poor hygiene [9], and tobacco and alcohol consumption [10], as well as lower access to health care [11-13].

Increasing social inequalities, the development of the private health sector, barriers to access to care among the poor [14,15] and the deterioration in the quality of the public health sector are all factors that may increase the differential vulnerability of tribal populations. However, Scheduled Tribes in India are demographically, culturally and economically heterogeneous, varying widely in terms of their population size, language, and the nature of their interactions with the rest of society [16,17]. Given that exclusion from social and economic opportunities varies in nature and magnitude [18,19], inequalities in health outcomes *between* tribal groups are likely. Yet most of the evidence on the health of Scheduled Tribes is available either at the aggregated level, failing to account for the diversity among Scheduled Tribe groups, or focuses on the health of a specific Scheduled Tribe, making it difficult to analyse inter-tribal inequalities in health.

Broad social and economic changes have taken place in India in the past 20 years, particularly with respect to affirmative action policies intended to benefit Scheduled Tribe members [20]. Land tenancy reforms initially showed impressive effects in the 1970s and 1980s in states such as Kerala, although progress has slowed in recent years [21]. Recent evidence suggests that Scheduled Tribes continue to be highly marginalised, remaining virtually landless, perceived as outsiders, not performing as well in school (higher dropout rate) and unable to take advantage of affirmative action policies [16]. Therefore, it is unclear whether health inequalities have decreased in recent years. A shift in the disease burden in India is also evident [22], underscoring the need for new evidence on the health divide using clinical measures of morbidity.

The primary aim of this study is to investigate the magnitude of the health divide, as measured by morbidity rates, between tribe and non-tribe populations, as well as between different tribal groups in a rural community in the southern state of Kerala. We also examine the extent to which health inequalities are explained by material deprivation.

## Methods

This study is part of a collaborative research program on inequalities in health and health care by the Centre for Development Studies and the University of Montreal in a single Gram Panchayat (the lowest territorial administrative unit) located in the northern district of Wayanad in Kerala [23]. Although Scheduled Tribe members account for only 1% of the population in Kerala State, there are clusters of tribal groups in some areas. The Panchayat in this study has a population of 16,110 of whom 28% have Scheduled Tribe affiliations. Three out of five Scheduled Tribe members in the Panchayat are Paniyas, who make up one-fifth of Kerala’s Scheduled Tribe population [24]. Historically, Paniyas were bonded labourers. Today they are predominantly landless agricultural labourers who tend to migrate for employment opportunities. Paniyas also face social marginalisation, high levels of impoverishment, and poor access to health care [25]. Small numbers of other tribal groups who have living standards similar to the general population are also found in the Panchayat. The Kuruchian constitutes the large majority of “other Scheduled Tribes” in this area (94% of non-Paniyas).

We draw on data from a health survey approved by the ethics committee of University of Montreal. Households were selected from a sampling frame with the complete list of households in the Gram Panchayat, developed by conducting a census. The list was stratified by location in 10 Panchayat wards and 17 percent of households were selected in each ward. A total of 543 households were selected through a circular systematic random sampling process; house numbers were arranged in a circular manner and every sixth household was selected. Over-sampling of the tribal population (n = 226) was done by surveying all households in a tribal colony where one household had been randomly sampled. Each individual belonging to the household was interviewed, with the exception of minors (<18 y). Members were asked to respond to a standard questionnaire and underwent a physical examination by trained health personnel. Four health conditions common in populations prior to an epidemiologic transition (underweight, anaemia, goitre and tuberculosis) and one health condition common in the post-transition period (hypertension) were assessed. These measures enabled us to explore various patterns of morbidity associated with nutritional deficiencies as well as the epidemiologic transition.

Weight and height were measured using standard methods [26]; underweight was defined as body mass index (BMI) < 18.5 kg/m^2^[27]. Pallor of the conjunctivae, tongue and nails was used to assess anaemia status [28]. Goitre was assessed by palpation of the thyroid gland [29]. Blood pressure was measured twice and the mean of the two readings was used for analyses. Hypertension was defined as the mean ≥140 and/or 90 mm Hg [30]. Suspected tuberculosis cases (cases eligible for sputum examination) were defined as either coughing for at least one week during the last four weeks and having had moderate fever at night in the last four weeks, or having had a positive tuberculosis test in the last 12 months [31].

To explore the health divide in adults, we compared individual age- and sex-standardised prevalence for morbidity measures. Standardised rates were computed using a direct method with the total study population as the standard population. Weights were assigned to generate representative estimates. Household caste or tribal affiliations were assigned to the three social groups conventionally used by the Government of India: Forward Castes (the most privileged), Other Backward Classes (a residual group of low castes), and the lowest ranked group, Scheduled Castes/Scheduled Tribes. The lowest group was further disaggregated by contrasting Paniyas with other Scheduled Tribe populations. To assess trends across age groups, analysis was done using three groups (18–30 vs*.* 31–59 vs*.* 60–96 y). The socioeconomic status of households was defined in three ways. The local administration classifies households as above (APL) or below (BPL) the poverty line using a three-step approach: households with land or asset holdings and a certain minimum income are considered APL; the poorest (destitute households, Maha Dalits, single women, households with disabled persons as breadwinner, households headed by minors) are considered BPL; and the remaining households are ranked based on a quality of life score (based on 13 parameters such as type of house, availability of clothes, sanitation, literacy, means of livelihood and indebtedness). In addition, we used household landholdings of more than 50 cents of land (100 cents is equal to 1 acre) and crowding (ratio of individuals to rooms ≥3) as indicators of socioeconomic status [32].

Multi-level logistic regression models were used to estimate the predicted prevalence of morbidity for each age and social group, controlling for other covariates. Estimates were based on the current distribution of poverty and education across caste-age groups and the local population sex ratio. Random effect models were used to adjust for clustering at the household level and provide robust standard error estimates.

To further explore the sources of the health gap between tribal and non-tribal groups, we used a Blinder-Oaxaca decomposition [33,34]. This technique enables us to quantify the part of the health gap due to group differences in the distribution of health determinants and the part due to differences in the effects of these determinants. The first component reflects differences in observable characteristics (endowments) between groups. The latter measures the part of the gap that remains unexplained after these characteristics are taken into account and is often seen as an indication of a discriminatory effect. In reality, the unexplained gap might reflect discrimination [35] or unequal treatment [36] of the groups, and also differences in omitted determinants of health [37]. The larger this remaining gap, the more likely discrimination and other unobservable factors play a role in explaining the health gap. We applied the method to decompose the gap in the prevalence of underweight, anaemia and goitre between tribal and non-tribal groups first, and then between Paniyas and Other Scheduled Tribes. Observable characteristics included in the models were: age, sex, education, poverty status, land ownership, occupation (wage labourer), housing condition (crowding) and access to safe drinking water. Estimates were obtained with statistical routines designed for non-linear outcomes (fairlie.ado and ndecompose.ado) [38]. We used Stata version 11 software for all analyses.

## Results

Health assessments were completed for a total of 1660 adults aged 18 years and above. Of the initial 543 households, 20 could not be located (3.7%), and 21 out of the 2418 individuals (0.9%) refused to participate. Characteristics of the sample are presented in Table 1 by social group. Inequalities are evident between Scheduled Tribe and non-tribe members for indicators of education and socioeconomic status. Over one-third of tribe members have no formal education compared to less than 10% of non-tribe members. The proportion of tribe members living in BPL households is two times higher than for non-tribe members. Among those who are employed, 74.1% of tribal and 40.7% of non-tribal members reported working as wage labourers. Inequalities within the Scheduled Tribes group are also observed. Lack of education is much higher among the Paniya compared to the other Scheduled Tribes. On average, 63.9% of Scheduled Tribe households own <50 cents land, but this ranges from 89.3% of Paniya to 50.2% of other Scheduled Tribes. Crowding is markedly higher among Paniya households compared to all other groups.

**Table 1:** Characteristics of study sample by social groups

	**Paniyas** **(a)**	**Other Scheduled Tribes** **(b)**	**Other Backward Classes** **(c)**	**Forward Castes** **(d)**	**Tribes** **(a+b)**	**Non-tribes** **(c+d)**	**All adults** **(a+b+c+d)**
**N**	**425**	**313**	**428**	**494**	**738**	**922**	**1660**
**Age group (years), %**							
**18–30**	40.2	39.0	36.4	25.3	39.4	30.6	33.3
**31–59**	45.6	47.3	49.1	55.3	46.7	52.3	50.6
**60+**	14.1	13.7	14.5	19.4	13.9	17.1	16.1
**Male, %**	41.2	49.5	46.0	49.8	46.6	48.0	47.6
**Never went to school, %**	53.6	26.8	14.7	2.4	36.3	8.2	17.0
**Household below poverty line (BPL), %**	95.0	77.3	49.1	35.4	83.5	41.9	54.9
**Wage labourers**^**1**^**, %**	97.1	60.6	57.1	26.4	74.1	40.7	53.4
**Household owns <50 cents land, %**	89.3	50.2	64.3	30.0	63.9	46.2	51.7
**Crowding (ratio individuals to rooms ≥3), %**	42.6	8.3	0.9	1.4	20.3	1.2	7.1

^1^Proportion among employed adults.

**Figure 1:**
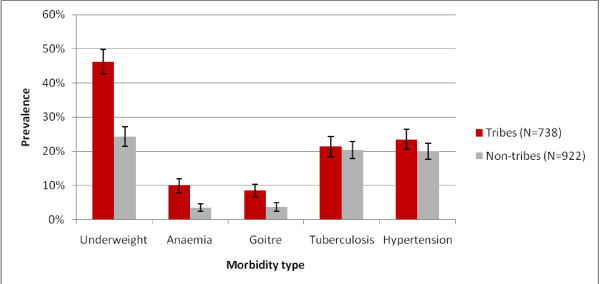
Morbidity across tribal and non-tribal groups (standardized by age and sex; error bars 95% CI).

**Figure 2:**
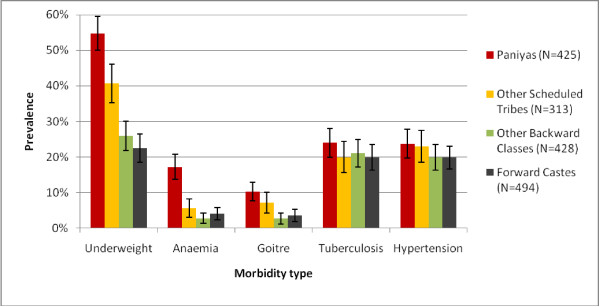
Morbidity across social groups (standardized by age and sex; error bars 95% CI).

Comparison of the age and sex standardised prevalence of morbidity between tribal and non-tribal groups (Figure 1) reveals a higher prevalence of underweight, anaemia and goitre among tribal members. When morbidity prevalence is further disaggregated by tribal and caste groups (Figure 2), a strong gradient is observed between social groups for underweight, anaemia and goitre, with the Paniya group having the highest prevalence, followed by the other Scheduled Tribes, and the lowest prevalence among Other Backward Class and Forward Caste members. Prevalence of suspected tuberculosis and hypertension is similar across the groups. (See Additional file 1 for non-standardised prevalence).

**Figure 3:**
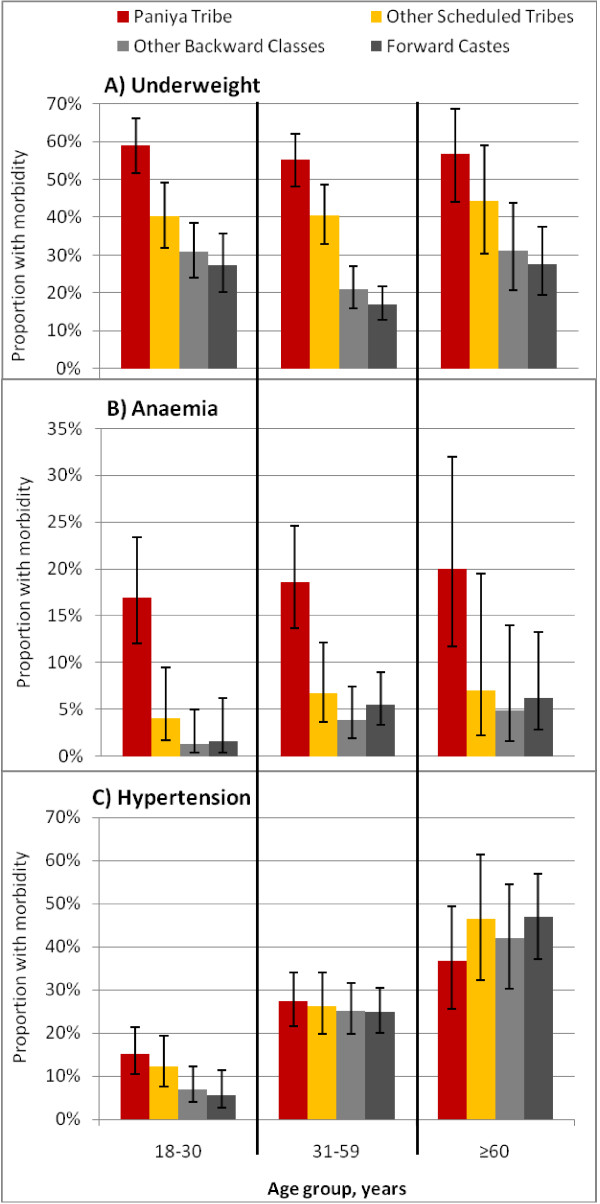
Morbidity prevalence by age and social group.

To examine trends among adults of different ages, morbidity was compared across social and age groups (Figure 3; goitre and tuberculosis are not shown owing to space constraints). For underweight and anaemia, a comparable social gradient is evident for all age groups, with the highest prevalence among the Paniya in each age group. Hypertension prevalence is positively associated with age group, as expected, for all social groups and is largely similar between social groups within the 31–59 y and 60–96 y age groups. However, within the youngest age group (18–30 y), a unique pattern is observed whereby Paniya members have a significantly higher prevalence of hypertension compared to young adults from Other Backward Class or Forward Caste groups.

The magnitude of excess morbidity was estimated by age group for each social group, with Forward Caste as the reference group (Table 2), adjusted for the local distribution of poverty and education across caste-age groups and the population sex ratio. Compared to a predicted prevalence of underweight of 27.1% (95% CI 18.8, 35.5) among Forward Caste members 18–30 y, the prevalence is 31 and 13 percentage points higher for Paniya and other Scheduled Tribe members, respectively. A similar pattern is seen in the two older age groups. The Paniya also have a significantly higher predicted prevalence of anaemia, between 11 and 14 percentage points, across all age groups. For hypertension, excess morbidity is only evident among Paniya adults 18–30 y, whose prevalence is 10 percentage points higher than among Forward Caste adults of the same age group (5.4%; 95% CI 1.6, 9.2). Hypertension in the two older age groups is similar across social groups, with the possible exception of older Paniya adults where a protective effect is suggested.

**Table 2:** Predicted excess morbidity (percentage points) by age and social group*

**Age group**	**Social group**	**Underweight**		**Anaemia**		**Hypertension**	
Young	FC [Ref]						
	OBC	+3.6	−7.7, 15.0	−0.3	−3.0, 2.3	+1.6	−3.8, 6.9
	Other ST	**+13.1**	1.0, 25.2	+2.5	−1.5, 6.4	+6.4	−0.5, 13.3
	Paniya	**+31.4**	20.0, 42.8	**+13.6**	**7.3, 19.9**	**+10.1**	**3.0, 17.3**
Middle age	FC [Ref]						
	OBC	+3.7	−4.2, 11.5	−2.0	−5.7, 1.6	+0.8	−7.4, 9.0
	Other ST	**+24.4**	**14.2, 34.6**	+0.7	−4.2, 5.5	+1.6	−7.4, 10.5
	Paniya	**+38.6**	**29.7, 47.4**	**+11.2**	**4.8, 17.6**	+2.6	−5.6, 10.9
Elderly	FC [Ref]						
	OBC	+3.6	−12.4, 19.6	−1.5	−8.9, 6.0	−5.9	−22.1, 10.3
	Other ST	+16.6	−1.4, 34.6	+0.3	−8.4, 9.0	−0.5	−20.7, 19.6
	Paniya	**+29.8**	**13.3, 46.4**	**+14.1**	**1.7, 26.5**	−11.1	−27.3, 5.2

* Predicted prevalence based on the current distribution of poverty and education across caste-age groups and local population sex ratio.

FC = Forward Castes; OBC = Other Backward Classes; Other ST = Other Scheduled Tribes.

**Figure 4:**
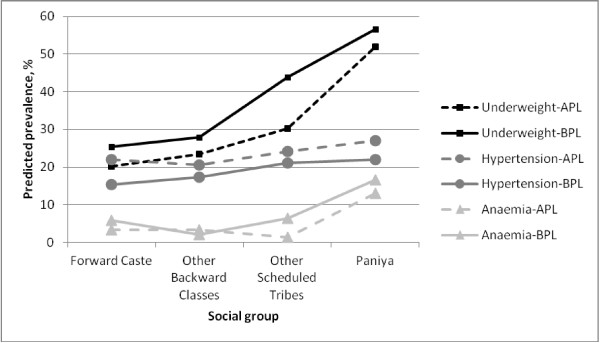
Predicted prevalence of underweight, anaemia and hypertension by social group and poverty status (based on the distribution of education across caste-poverty groups and the local population sex ratio & age group).

Figure 4 presents the predicted prevalence of morbidity for the poor (BPL) and non-poor (APL) within each social group, controlling for other covariates. These results highlight, again, the magnitude of the social gradient in health, but more importantly, they show that this social gradient holds for both the poor and the non-poor. There is no evidence in this context that poverty alone provides a satisfactory explanation for the existence of the health divide between tribes and non-tribes. This is further supported by the Blinder-Oaxaca decomposition of the health gaps; Table 3 presents the overall results showing the proportion of the health gap explained by endowments (detailed results for the relative contribution of each of the covariates are presented in Additional file 2). Even after controlling for a large set of social determinants of health, an important health gap (difference in predicted prevalence) remains between tribes and non-tribes as well as between Paniyas and other Scheduled Tribes. For underweight and goitre, differences in endowments between tribes and non-tribes explain 54% and 42% of the health gap respectively; roughly half of the health gap remains unexplained. The results are similar for these two outcomes in the model comparing the Paniyas to other Scheduled Tribes. The situation is different for anaemia. The health divide between tribes and non-tribes for anaemia is largely explained by the inequality in the distribution of endowments between the two groups (86% of the health gap). But when contrasting the Paniyas with other tribal groups, the gap remains largely unexplained after taking into consideration the distribution of the socioeconomic factors. Sickle cell anaemia, a hereditary disease that is prevalent among the Paniya [39], may explain this difference in predicted prevalence of anaemia from the other tribes. Nonetheless, as expected, the analysis of the respective contributions of the major socioeconomic factors shows that the concentration of poverty and lack of education among the Scheduled Tribes contributes significantly to the excess morbidity observed in this group compared to the general population. The results are less clear for the health gap between Paniyas and other tribes.

**Table 3:** Decomposition of the health gap between (i) tribal and non tribal populations; and (ii) Paniya and Other Scheduled Tribe populations

Outcome	**Tribal vs Non Tribal populations**			**Paniya vs Other ST populations**		
	Health Gap^‡^	Diff due to endowments	Diff due to social status	Health Gap^‡^	Diff due to endowments	Diff due to social status
Underweight	0.233	0.126	0.107 (46%)	0.164	0.076	0.088 (54%)
Anaemia	0.050	0.021	0.029 (58%)	0.045	0.025	0.022 (49%)
Goitre	0.063	0.054	0.009 (14%)	0.124	−0.001	0.125 (100%)

^†^ Model covariates: age 18–30, age 31–59, sex, education, poverty (BPL), land ownership, wage labourer, crowding, water quality.

^‡^ Difference in predicted prevalence between the advantaged and the disadvantaged group.

## Discussion

Health inequalities are evident in a rural district of Kerala, with Scheduled Tribe members bearing a higher burden of morbidity compared to non-tribe groups. However, not all indigenous groups are equally disadvantaged. The largest tribal group, the Paniya, have higher unmet health needs compared to other Scheduled Tribes in this area, specifically with respect to underweight, anaemia and goitre. Comparison of morbidity across age groups reveals that this social gradient of inequality is apparent across generations. In contrast to levels observed in older adults, higher hypertension levels among young Paniya adults compared to their peers in other social groups point to a greater double burden of disease in this subgroup. Although tribal groups have higher levels of poverty, stratifying morbidity by household economic status does not change the gradient observed.

### Strengths and limitations of study

This is the first study in Kerala, to our knowledge, to explore the extent of social inequalities in health between indigenous and non-indigenous people based on multiple clinical markers of morbidity. This allows us to quantify the vulnerability of indigenous groups across the spectrum of deteriorating health and disease, both in terms of morbidity resulting from the classical conditions of deprivation associated with poverty (anaemia, goitre, undernutrition and tuberculosis) and that associated with the epidemiologic transition (hypertension as a marker of cardiovascular risk). The limitations of our study lie first of all in the capacity to explore in greater depth how each disease process evolved. A more systematic study of each morbidity type would require biological and clinical investigations that go beyond the current population survey approach and objectives of the study. Secondly, none of the indicators assessed are able to reflect the situation unequivocally. Genetic differences between ethnic groups contribute to differences in attained adult height that may bias the validity of BMI <18.5 kg/m^2^ as a measure of underweight in this area; however, models including only adults with a height over 150 cm provided similar results. Anaemia was measured using clinical indicators associated with severe anaemia and may be more prevalent among the Paniya than other groups for genetic reasons, such as a higher prevalence of sickle cell disease [39,40]. Nutritional deficiencies are not the only causative factor of goitre. Different survival rates across groups may have also moderated the gaps observed, given the lower life expectancy for the Paniyas and other Scheduled Tribes. Nonetheless the convergence of results and consistency of the patterns observed are reassuring and support the central hypothesis about the vulnerability of indigenous people, in particular the Paniyas.

### Comparison with other studies

Heightened vulnerability of Scheduled Tribes to underweight [41,42] and anaemia [6] has been shown in other studies at a national level. While the risk of underweight was associated with lower socioeconomic status in those studies and ours, the odds of being underweight were still nearly twice as high for tribe compared to non-tribe members in our study, independent of their household poverty status. Furthermore, despite the widespread improvement in education and other social determinants of health and nutrition, there is no evidence in our sample of a reduction in underweight prevalence among younger adults from any social group. This is consistent with findings from a study comparing underweight in Indian adults between national surveys in 1998–99 and 2005–06 [41]. During this period of time, underweight prevalence in women decreased by only three percentage points and no age-related changes in underweight prevalence were observed.

Despite the epidemiological transition taking place in Kerala State [43,44] and the potential for heightened sensitivity to lifestyle-related chronic disease risk factors as a result of childhood undernutrition [45,46], no major shift in disease burden was observed in our sample. Hypertension prevalence estimates in rural areas of India ranged from 10% to 20% in earlier studies [47], similar to the levels found in this study, but have been as high as 42% in a more recent study in Kerala [43]. The higher hypertension levels observed among young Paniya adults suggests a higher level of vulnerability to post-transition diseases in these adults at the lower rung of society, although we did not assess in this study the potential contribution of genetic factors. Stress or psychosocial adversity in these individuals may contribute to this vulnerability, as younger Paniya members have not experienced improved life and work opportunities, despite benefiting from increased education opportunities [16]. Chronic stress associated with social position has biological effects that may contribute specifically to social gradients in risk of coronary disease and other morbidity [48]. Psychosocial factors also directly affect health related behaviours such as smoking, diet, alcohol consumption and physical activity, which also play an important role in blood pressure regulation [49]. Further research into the specific factors contributing to hypertension in this subgroup is needed.

Overall, the accumulation of health deficits in the Paniya group is indicative of their heightened vulnerability in terms of high exposure to health risks and barriers to accessing health care [50]. It is also consistent with the Paniyas’ own perceptions as voiced in focus group discussions, in which numerous vulnerability traps related to a range of risk factors were identified, including poor health, landlessness, poverty, exposure to harsh environmental conditions (e.g. floods), alcohol use, colony isolation, and education deficits [25,51]. The consistency between our survey findings and the views of the Paniyas themselves increases our confidence in the robustness of the results.

Although caste-related differences in Kerala may be lower than in other parts of India, the results of our study suggest that this social determinant still underlies overall inequality [52]. By disaggregating the analyses by groups that mirror the social caste hierarchy, we observed a consistent health gradient across these groups. Individuals with Forward Caste affiliations are in better health compared to Other Backward Classes, who in turn are in better health than Scheduled Tribes, with Paniyas having the poorest health. A similar gradient in risk factors for ill health across the four groups was also observed (data not shown). An earlier study among women in this area also showed inequalities across caste groups in women’s perceived health, measured by self-reported health and limitations in daily activity [53].

Excluding the case of anaemia, roughly half of the health gap between tribes and non-tribes remains unexplained by differences in endowments. Our results correspond with those of Das [36] and Borooah [54] on the inequalities of poverty in India. In their respective studies, the authors found that the greater part of the difference in poverty rates between Scheduled Tribes and the general population was attributable to differences in coefficients rather than endowments; a result seen as “a likely indication of discrimination” [36]. In a recent study aimed at decomposing malnutrition inequalities in India, Van de Poel and Speybroeck [55] found that more than one-third of the malnutrition gap was attributable to differences in the effects of health determinants after controlling for a large set of explanatory variables.

Can the observed health gradient be explained solely by the concentration of poverty among tribal groups? Our results suggest not and are in contrast to others that suggest caste is diminishing in its importance in determining social status and associated health inequalities in India [56]. A recent study on mortality showed mixed results, with evidence for attenuation of caste differences when adjusted for living standards in the working age group (19–64 y) but a strong influence of caste in younger age groups, even after controlling for standard of living [3]. In our study of adults, the health gradient between social groups was still evident among those of similar economic status, indicating that health inequalities are rooted in social structures much deeper than material deprivation. The Blinder Oaxaca decomposition supports this position; differences in the effects of health determinants appear to play an important role. The unexplained part of the health gap reflects differences in the processes that generate health outcomes across social groups. It is likely that a significant part of this gap is driven by discriminatory practices and differential rates of returns on endowments for tribal and non tribal populations, especially since differences in endowments between indigenous and non-indigenous groups (poverty status, occupation, education, housing conditions, etc.) are themselves largely attributable to past exclusion and discrimination practices [36].

## Conclusions

In this paper, we used clinical measures of morbidity, supporting other studies that found a social gradient in mortality and self perceived measures of health [3,53]. Our findings highlight that this gradient is not solely explained by differences in material deprivation, suggesting that other factors, such as social exclusion and caste discrimination, are also important [57]. There is a need for future research to systematically test the role of discrimination in the health of Scheduled Tribes and other marginalised groups in India.

Even in the egalitarian state of Kerala, the health of Scheduled Tribes continues to lag behind other social groups. Moreover, significant health inequalities also exist within these tribal populations, with one tribe having very distinct needs compared to others in our study context. This necessitates moving beyond the standard approach of categorising all tribes as one equally disadvantaged group. Policies and programmes designed to benefit the Scheduled Tribes need to be tailored to not only promote the well-being of tribes in general but also to target the specific needs of each tribe, needs that differ both in nature and severity. Furthermore, these policies “cannot be limited to enhancing endowments” [36] but must also enhance the capacity of the disadvantaged to equally take advantage of these endowments. Improving the health of Scheduled Tribes will likely require multi-pronged efforts that try to address the very roots of inequality, discrimination and social injustice that constrains their opportunities to live healthy lives.

## Competing interests

The authors’ declare that they have no competing interests.

## Authors’ contributions

SH, KM and DN conceived the study. SH and KS wrote the first draft, and the paper was edited by all authors. SH, GM, and KS did the statistical analysis. All authors interpreted the data, critically revised the draft, and gave approval for the paper to be published. All authors had full access to all of the data in the study and can take responsibility for the integrity of the data and the accuracy of the data analysis. SH is guarantor. All authors have read and approved the final manuscript.

## Pre-publication history

The pre-publication history for this paper can be accessed here:

http://www.biomedcentral.com/1471-2458/12/390/prepub

## Supplementary Material

Additional file 1 Tables presenting non-standardized morbidity prevalence across groups. Table A: Morbidity across tribal and non-tribal groups (non-standardized). Table B: Morbidity across social groups (non-standardized). This additional file provides two supplementary tables presenting the non-standardized prevalence of morbidity for tribal and non-tribal groups (Table A) and for all social groups (Table B). Click here for file

Additional file 2 Oaxaca-Blinder Decomposition Detailed Regression Results. This additional file provides the detailed results for each Oaxaca-Blinder decomposition regression model, from which the results in Table 3 were summarized. Click here for file
