# Supplementary material for: “Health divide” between indigenous and non-indigenous populations in Kerala, India: Population based study
Source: BMC Public Health. 2012 May 29;12:390. doi: 10.1186/1471-2458-12-390 (PMC3441884; doi:10.1186/1471-2458-12-390)
Supplement: Additional file 1 — Tables presenting non-standardized morbidity prevalence across groups. Table A: Morbidity across tribal and non-tribal groups (non-standardized). Table B: Morbidity across social groups (non-standardized). This additional file provides two supplementary tables presenting the non-standardized prevalence of morbidity for tribal and non-tribal groups (Table A) and for all social groups (Table B). [file 1471-2458-12-390-S1.doc]

## Tables presenting non-standardized morbidity prevalence across groups

## Table A: Morbidity across tribal and non-tribal groups (non-standardized)

|  | **Tribes (N=738)** | **Non-tribes (N=922)** | **All (n=1660)** |
| --- | --- | --- | --- |
| Underweight | 46.6 [42.7, 50.5] | 23.7 [21.0, 26.6] | 30.9 [28.6, 33.2] |
| Anaemia | 10.1 [8.2, 12.5] | 3.9 [2.8, 5.4] | 5.8 [4.8, 7.0] |
| Goitre | 8.7 [6.8, 11.1] | 3.7 [2.6, 5.1] | 5.3 [4.3, 6.4] |
| Tuberculosis | 21.7 [18.6, 25.1] | 21.7 [19.1, 24.5] | 21.7 [19.6, 23.9] |
| Hypertension | 23.7 [20.5, 27.2] | 22.8 [20.2, 25.6] | 23.0 [21.0, 25.3] |

## Table B: Morbidity across social groups (non-standardized)

|  | **Paniyas**  **(N=425)** | **Other Scheduled Tribes (N=313)** | **Other Backward Classes (N=428)** | **Forward Castes**  **(N=494)** |
| --- | --- | --- | --- | --- |
| Underweight | 56.9 [52.2, 61.6] | 40.9 [35.6, 46.4] | 26.0 [22.1, 30.4] | 21.6 [18.2, 25.5] |
| Anaemia | 18.1 [14.7, 22.1] | 5.8 [3.7, 9.0] | 3.0 [1.8, 5.2] | 4.7 [3.1, 6.9] |
| Goitre | 11.8 [9.0, 15.2] | 7.0 [4.7, 10.5] | 2.8 [1.6, 4.9] | 4.5 [3.0, 6.7] |
| Tuberculosis | 24.4 [20.5, 28.9] | 20.2 [16.1, 25.1] | 21.6 [17.8, 25.8] | 21.7 [18.2, 25.7] |
| Hypertension | 23.8 [20.0, 28.1] | 23.6 [19.3, 28.7] | 21.0 [17.4, 25.2] | 24.3 [20.7, 28.3] |
