# Supplementary material for: “Health divide” between indigenous and non-indigenous populations in Kerala, India: Population based study
Source: BMC Public Health. 2012 May 29;12:390. doi: 10.1186/1471-2458-12-390 (PMC3441884; doi:10.1186/1471-2458-12-390)
Supplement: Additional file 2 — Oaxaca-Blinder Decomposition Detailed Regression Results. This additional file provides the detailed results for each Oaxaca-Blinder decomposition regression model, from which the results in Table 3 were summarized. [file 1471-2458-12-390-S2.doc]

**Additional File 2 – Oaxaca-Blinder Decomposition Detailed Regression Results**

**Tribal vs. non tribal populations**

**Results for Underweight**

Logistic regression Number of obs = 662

Wald chi2(9) = 46.55

Prob > chi2 = 0.0000

Log pseudolikelihood = -428.12721 Pseudo R2 = 0.0631

------------------------------------------------------------------------------

| Robust

underweight | Coef. Std. Err. z P>|z| [95% Conf. Interval]

-------------+----------------------------------------------------------------

female | .4051613 .2005391 2.02 0.043 .0121119 .7982108

age18-30 | .8054987 .3715825 2.17 0.030 .0772105 1.533787

age31-59 | .4275345 .3588923 1.19 0.234 -.2758815 1.130951

Not educated | .8564505 .2128835 4.02 0.000 .4392066 1.273694

Poor (BPL) | .5530632 .2839831 1.95 0.051 -.0035334 1.10966

No land &| .2724758 .2187335 1.25 0.213 -.1562341 .7011856

Crowd * | .3509693 .2056771 1.71 0.088 -.0521505 .754089

No_waterqual#| -.4374736 .3211809 -1.36 0.173 -1.066977 .1920294

N_wagelabor | -.1570052 .2002227 -0.78 0.433 -.5494346 .2354242

_cons | -2.242137 .5142587 -4.36 0.000 -3.250066 -1.234209

------------------------------------------------------------------------------

No land & : own less than 50 cents of land

Crowd *  : more than 3 individuals per room

No_waterqual# : source of drinking water of poor quality

N_wagelabor : Not a wage labourer

Non-linear decomposition by nontribe2 (G)

Number of obs = 1474

N of obs G=0 = 662

N of obs G=0 = 812

Pr(Y!=0|G=0) = .46212056

Pr(Y!=0|G=1) = .22951821

Difference = .23260235

Total explained = .12607562

------------------------------------------------------------------------------

underweight | Coef. Std. Err. z P>|z| [95% Conf. Interval]

-------------+----------------------------------------------------------------

female | .001904 .0017582 1.08 0.279 -.001542 .0053501

age18-30 | .0149224 .0080249 1.86 0.063 -.0008061 .03065

age31-59 | -.0069891 .0066389 -1.05 0.292 -.020001 .0060228

Not educated | .0540669 .0130755 4.13 0.000 .0284395 .0796944

Poor (BPL) | .0479726 .0235755 2.03 0.042 .0017654 .0941798

No land &| .0111001 .0089651 1.24 0.216 -.0064712 .0286713

Crowd * | .0153446 .009066 1.69 0.091 -.0024244 .0331135

No_waterqual#| -.0025968 .0021705 -1.20 0.232 -.0068509 .0016574

N_wagelabor | -.008779 .0112265 -0.78 0.434 -.0307825 .0132244

------------------------------------------------------------------------------

**Results for Anemia**

Logistic regression Number of obs = 662

Wald chi2(9) = 37.13

Prob > chi2 = 0.0000

Log pseudolikelihood = -194.33163 Pseudo R2 = 0.0937

------------------------------------------------------------------------------

| Robust

anemia | Coef. Std. Err. z P>|z| [95% Conf. Interval]

-------------+----------------------------------------------------------------

female | .7805719 .3298679 2.37 0.018 .1340427 1.427101

age18-30 | .0839174 .5768694 0.15 0.884 -1.046726 1.214561

age31-59 | .0270883 .5446873 0.05 0.960 -1.040479 1.094656

Not educated | .8370992 .3435398 2.44 0.015 .1637736 1.510425

Poor (BPL) | 1.337698 .596876 2.24 0.025 .1678428 2.507554

No land &| -.2608952 .3339656 -0.78 0.435 -.9154557 .3936653

Crowd * | .3068788 .2963228 1.04 0.300 -.2739033 .8876609

No_waterqual#| .9559973 .3878558 2.46 0.014 .195814 1.716181

N_wagelabor | .1714345 .2796712 0.61 0.540 -.376711 .71958

_cons | -5.178385 .8714711 -5.94 0.000 -6.886436 -3.470333

------------------------------------------------------------------------------

No land & : own less than 50 cents of land

Crowd *  : more than 3 individuals per room

No_waterqual# : source of drinking water of poor quality

N_wagelabor : Not a wage labourer

Decomposition replications (100)

----+--- 1 ---+--- 2 ---+--- 3 ---+--- 4 ---+--- 5

.................................................. 50

.................................................. 100

Non-linear decomposition by nontribe2 (G)

Number of obs = 1474

N of obs G=0 = 662

N of obs G=0 = 812

Pr(Y!=0|G=0) = .09946374

Pr(Y!=0|G=1) = .03675133

Difference = .06271241

Total explained = .05403076

------------------------------------------------------------------------------

anemia | Coef. Std. Err. z P>|z| [95% Conf. Interval]

-------------+----------------------------------------------------------------

female | .0012417 .0024966 0.50 0.619 -.0036516 .0061349

age18-30 | .0002587 .0026427 0.10 0.922 -.0049209 .0054382

age31-59 | -.0000934 .0025714 -0.04 0.971 -.0051333 .004946

Not educated | .0217422 .0090319 2.41 0.016 .0040399 .0394445

Poor (BPL) | .02215 .0078096 2.84 0.005 .0068435 .0374564

No land &| -.00321 .004913 -0.65 0.514 -.0128394 .0064193

Crowd * | .0049278 .0052482 0.94 0.348 -.0053584 .015214

No_waterqual#| .0034358 .0021475 1.60 0.110 -.0007733 .007645

N_wagelabor | .0030907 .005328 0.58 0.562 -.0073521 .0135335

------------------------------------------------------------------------------

**Results for Goitre**

Logistic regression Number of obs = 662

Wald chi2(9) = 70.10

Prob > chi2 = 0.0000

Log pseudolikelihood = -170.46258 Pseudo R2 = 0.1468

------------------------------------------------------------------------------

| Robust

goitre | Coef. Std. Err. z P>|z| [95% Conf. Interval]

-------------+----------------------------------------------------------------

female | 2.370863 .442248 5.36 0.000 1.504072 3.237653

age18-30 | .7206812 .9092163 0.79 0.428 -1.06135 2.50271

age31-59 | .0620777 .8660954 0.07 0.943 -1.635438 1.759593

Not educated | -.2680925 .3542606 -0.76 0.449 -.9624306 .4262456

Poor (BPL) | .3675467 .4864126 0.76 0.450 -.5858045 1.320898

No land &| -.6902662 .3639024 -1.90 0.058 -1.403502 .0229694

Crowd * | -.071813 .3222867 -0.22 0.824 -.7034832 .5598573

No_waterqual#| .2874902 .489609 0.59 0.557 -.6721259 1.247106

N_wagelabor | .9810841 .3193062 3.07 0.002 .3552555 1.606913

_cons | -7.057207 1.277045 -5.53 0.000 -9.560169 -4.554246

------------------------------------------------------------------------------

No land & : own less than 50 cents of land

Crowd *  : more than 3 individuals per room

No_waterqual# : source of drinking water of poor quality

N_wagelabor : Not a wage labourer

Decomposition replications (100)

----+--- 1 ---+--- 2 ---+--- 3 ---+--- 4 ---+--- 5

.................................................. 50

.................................................. 100

Non-linear decomposition by nontribe2 (G)

Number of obs = 1474

N of obs G=0 = 662

N of obs G=0 = 812

Pr(Y!=0|G=0) = .08968663

Pr(Y!=0|G=1) = .03926123

Difference = .05042539

Total explained = .02063092

------------------------------------------------------------------------------

goitre | Coef. Std. Err. z P>|z| [95% Conf. Interval]

-------------+----------------------------------------------------------------

female | .0006816 .0050907 0.13 0.893 -.0092961 .0106592

age18-30 | .0047921 .0110673 0.43 0.665 -.0168995 .0264837

age31-59 | -.0003932 .0082562 -0.05 0.962 -.0165751 .0157886

Not educated | -.0053687 .0074414 -0.72 0.471 -.0199537 .0092162

Poor (BPL) | .0094967 .0119298 0.80 0.426 -.0138853 .0328787

No land &| -.0094624 .0070262 -1.35 0.178 -.0232335 .0043087

Crowd * | -.0009126 .00416 -0.22 0.826 -.0090661 .0072408

No_waterqual#| .0009189 .001856 0.50 0.621 -.0027188 .0045567

N_wagelabor | .0210443 .0087104 2.42 0.016 .0039721 .0381165

------------------------------------------------------------------------------

.

**Paniya vs. Other Scheduled Tribe populations**

**Results for Underweight**

Logistic regression Number of obs = 378

Wald chi2(9) = 40.58

Prob > chi2 = 0.0000

Log pseudolikelihood = -235.77151 Pseudo R2 = 0.0876

------------------------------------------------------------------------------

| Robust

underweight | Coef. Std. Err. z P>|z| [95% Conf. Interval]

-------------+----------------------------------------------------------------

female | 1.029415 .2504442 4.11 0.000 .5385537 1.520277

age18-30 | .8532471 .4940292 1.73 0.084 -.1150322 1.821527

age31-59 | .4507315 .4742366 0.95 0.342 -.4787552 1.380218

Not educated | .837515 .2477428 3.38 0.001 .3519481 1.323082

Poor (BPL) | .0159377 .4801398 0.03 0.974 -.925119 .9569943

No land &| .0979674 .3634306 0.27 0.787 -.6143434 .8102783

Crowd * | .0609207 .2256137 0.27 0.787 -.381274 .5031154

No_waterqual#| -.3554803 .3622148 -0.98 0.326 -1.065408 .3544476

N_wagelabor | -.1498982 .2593965 -0.58 0.563 -.6583059 .3585095

_cons | -2.368522 .8161503 -2.90 0.004 -3.968147 -.7688966

------------------------------------------------------------------------------

No land & : own less than 50 cents of land

Crowd *  : more than 3 individuals per room

No_waterqual# : source of drinking water of poor quality

N_wagelabor : Not a wage labourer

Decomposition replications (100)

----+--- 1 ---+--- 2 ---+--- 3 ---+--- 4 ---+--- 5

.................................................. 50

.................................................. 100

Non-linear decomposition by pan (G) Number of obs = 662

N of obs G=0 = 378

N of obs G=0 = 284

Pr(Y!=0|G=0) = .56878307

Pr(Y!=0|G=1) = .40492958

Difference = .16385349

Total explained = .07642709

------------------------------------------------------------------------------

underweight | Coef. Std. Err. z P>|z| [95% Conf. Interval]

-------------+----------------------------------------------------------------

female | .0234164 .0061153 3.83 0.000 .0114307 .0354021

age18-30 | .0016717 .0060613 0.28 0.783 -.0102083 .0135516

age31-59 | .0005428 .0048404 0.11 0.911 -.0089442 .0100299

Not educated | .0508608 .0150919 3.37 0.001 .0212812 .0804404

Poor (BPL) | .0006161 .0187019 0.03 0.974 -.0360389 .0372711

No land &| .0079194 .0295928 0.27 0.789 -.0500815 .0659203

Crowd * | .0045179 .0168018 0.27 0.788 -.028413 .0374488

No_waterqual#| -.0031328 .0035241 -0.89 0.374 -.0100398 .0037742

N_wagelabor | -.0088182 .0154423 -0.57 0.568 -.0390845 .0214481

------------------------------------------------------------------------------

**Results for Anaemia**

Logistic regression Number of obs = 378

Wald chi2(9) = 16.73

Prob > chi2 = 0.0532

Log pseudolikelihood = -169.84769 Pseudo R2 = 0.0465

------------------------------------------------------------------------------

| Robust

anemia | Coef. Std. Err. z P>|z| [95% Conf. Interval]

-------------+----------------------------------------------------------------

female | .7833876 .3420745 2.29 0.022 .1129339 1.453841

age18-30 | -.1089882 .5953509 -0.18 0.855 -1.275855 1.057878

age31-59 | -.1427789 .5798078 -0.25 0.805 -1.279181 .9936236

Not educated | .4696499 .2928057 1.60 0.109 -.1042387 1.043539

Poor (BPL) | .4046565 .637949 0.63 0.526 -.8457005 1.655014

No land &| -.5926835 .4071475 -1.46 0.145 -1.390678 .205311

Crowd * | -.0158151 .2757015 -0.06 0.954 -.5561802 .5245499

No_waterqual#| .5713146 .417044 1.37 0.171 -.2460767 1.388706

N_wagelabor | -.1974791 .3060709 -0.65 0.519 -.7973671 .4024089

_cons | -2.770914 .9776652 -2.83 0.005 -4.687103 -.8547255

------------------------------------------------------------------------------

No land & : own less than 50 cents of land

Crowd *  : more than 3 individuals per room

No_waterqual# : source of drinking water of poor quality

N_wagelabor : Not a wage labourer

Decomposition replications (100)

----+--- 1 ---+--- 2 ---+--- 3 ---+--- 4 ---+--- 5

.................................................. 50

.................................................. 100

Non-linear decomposition by pan (G) Number of obs = 662

N of obs G=0 = 378

N of obs G=0 = 284

Pr(Y!=0|G=0) = .17989418

Pr(Y!=0|G=1) = .05633803

Difference = .12355615

Total explained = -.00054287

------------------------------------------------------------------------------

anemia | Coef. Std. Err. z P>|z| [95% Conf. Interval]

-------------+----------------------------------------------------------------

female | .0101233 .0050454 2.01 0.045 .0002345 .020012

age18-30 | -.000258 .0048643 -0.05 0.958 -.0097919 .0092759

age31-59 | .0000198 .0049207 0.00 0.997 -.0096245 .0096641

Not educated | .0184014 .0125 1.47 0.141 -.0060982 .042901

Poor (BPL) | .0099182 .0146933 0.68 0.500 -.0188801 .0387165

No land &| -.0341413 .0265065 -1.29 0.198 -.086093 .0178104

Crowd * | -.0007863 .013786 -0.06 0.955 -.0278064 .0262337

No_waterqual#| .0036721 .0037134 0.99 0.323 -.0036061 .0109503

N_wagelabor | -.007155 .0115395 -0.62 0.535 -.0297721 .015462

------------------------------------------------------------------------------

**Results for Goitre**

Logistic regression Number of obs = 378

Wald chi2(9) = 31.23

Prob > chi2 = 0.0003

Log pseudolikelihood = -116.40061 Pseudo R2 = 0.1564

------------------------------------------------------------------------------

| Robust

goitre | Coef. Std. Err. z P>|z| [95% Conf. Interval]

-------------+----------------------------------------------------------------

female | 3.08573 .7377655 4.18 0.000 1.639736 4.531724

age18-30 | .9292306 1.121905 0.83 0.408 -1.269662 3.128123

age31-59 | .566349 1.121841 0.50 0.614 -1.63242 2.765118

Not educated | -.5902932 .3956495 -1.49 0.136 -1.365752 .1851655

Poor (BPL) | -.4058137 .6219213 -0.65 0.514 -1.624757 .8131296

No land &| .5872091 .6650289 0.88 0.377 -.7162236 1.890642

Crowd * | -.0279322 .3406057 -0.08 0.935 -.695507 .6396427

No_waterqual#| -.0830542 .5296114 -0.16 0.875 -1.121073 .954965

N_wagelabor | .2836141 .3452953 0.82 0.411 -.3931522 .9603805

_cons | -8.315536 1.8434 -4.51 0.000 -11.92853 -4.702539

------------------------------------------------------------------------------

No land & : own less than 50 cents of land

Crowd *  : more than 3 individuals per room

No_waterqual# : source of drinking water of poor quality

N_wagelabor : Not a wage labourer

Decomposition replications (100)

----+--- 1 ---+--- 2 ---+--- 3 ---+--- 4 ---+--- 5

.................................................. 50

.................................................. 100

Non-linear decomposition by pan (G) Number of obs = 662

N of obs G=0 = 378

N of obs G=0 = 284

Pr(Y!=0|G=0) = .11904762

Pr(Y!=0|G=1) = .07394366

Difference = .04510396

Total explained = .02536233

------------------------------------------------------------------------------

vthyroid_d~y | Coef. Std. Err. z P>|z| [95% Conf. Interval]

-------------+----------------------------------------------------------------

female | .0175648 .0077762 2.26 0.024 .0023237 .0328059

age18-30 | .0051635 .0200115 0.26 0.796 -.0340583 .0443853

age31-59 | -.0034414 .0166298 -0.21 0.836 -.0360353 .0291525

Not educated | -.0099422 .0079737 -1.25 0.212 -.0255704 .0056861

Poor (BPL) | -.0070548 .0124064 -0.57 0.570 -.0313709 .0172612

No land &| .017143 .0174852 0.98 0.327 -.0171273 .0514133

Crowd * | -.0008737 .0107725 -0.08 0.935 -.0219874 .02024

No_waterqual#| -.0003169 .0023441 -0.14 0.892 -.0049112 .0042774

N_wagelabor | .0059202 .0076352 0.78 0.438 -.0090444 .0208848

------------------------------------------------------------------------------
